# C-Type Lectin-20 Interacts with ALP1 Receptor to Reduce Cry Toxicity in *Aedes aegypti*

**DOI:** 10.3390/toxins10100390

**Published:** 2018-09-25

**Authors:** Khadija Batool, Intikhab Alam, Guohui Zhao, Junxiang Wang, Jin Xu, Xiaoqiang Yu, Enjiong Huang, Xiong Guan, Lingling Zhang

**Affiliations:** 1 State Key Laboratory of Ecological Pest Control for Fujian and Taiwan Crops, College of Life Sciences, Key Lab of Biopesticides and Chemical Biology, MOE, Fujian Agriculture and Forestry University, Fuzhou 350002, Fujian, China; khadijabatoolali@gmail.com (K.B.); Zhaogh1019@outlook.com (G.Z.); junxiangwang@outlook.com (J.W.); xujinfafu@outlook.com (J.X.); yux@umkc.edu (X.Y.); guanxfafu@126.com (X.G.); 2 Key Laboratory of Genetics, Breeding and Comprehensive Utilization of Crops, Ministry of Education, College of Crop Science, Fujian Agriculture and Forestry University, Fuzhou 350002, Fujian, China; intikhabalam2013@gmail.com; 3 Division of Cell Biology and Biophysics, University of Missouri-Kansas City, Kansas City, MO 64110, USA; 4 Fujian International Travel Healthcare Center, Fuzhou 350001, Fujian, China; enjiong@163.com

**Keywords:** *Aedes aegypti*, C-type lectin, *Bacillus thuringiensis* Cry11A, alkaline phosphatase, toxicity

## Abstract

*Aedes aegypti* is a crucial vector for human diseases, such as yellow fever, dengue, chikungunya, and Zika viruses. Today, a major challenge throughout the globe is the insufficient availability of antiviral drugs and vaccines against arboviruses, and toxins produced by *Bacillus thuringiensis* (Bt) are still used as biological agents for mosquito control. The use of Cry toxins to kill insects mainly depends on the interaction between Cry toxins and important toxin receptors, such as alkaline phosphatase (ALP). In this study, we investigated the function of *A. aegypti* C-type lectin-20 (CTL-20) in the tolerance of Cry toxins. We showed that recombinant CTL-20 protein interacted with both Cry11Aa and ALP1 by the Far-Western blot and ELISA methods, and CTL-20 bound to *A. aegypti* larval brush border membrane vesicles (BBMVs). Binding affinity of CTL-20 to ALP1 was higher than that of Cry11Aa to ALP1. Furthermore, the survival rate of *A. aegypti* larvae fed with Cry11Aa toxin mixed with recombinant CTL-20 fusion protein was significantly increased compared with that of the control larvae fed with Cry11Aa mixed with thioredoxin. Our novel results suggest that midgut proteins like CTLs may interfere with interactions between Cry toxins and toxin receptors by binding to both Cry toxins and receptors to alter Cry toxicity.

## 1. Introduction

The mosquito *Aedes aegypti* is one of the serious diseases-causing vectors, closely associated with tropical areas of the world and native to Africa [1]. *A. aegypti* transmits rapidly emerging arboviruses, including yellow fever, dengue [2], chikungunya, and Zika viruses that spread widely throughout the world [3,4,5,6]. Clinically, dengue virus is the most important arbovirus, infecting 390 million patients every year as a result of the existence and complexity of different serotypes [7,8,9]. During the last 40 years, there has been an alarming increase of dengue virus of almost 30-fold, recorded in 90 countries including Australia, Southern Europe, and United States [10]. Currently, because of insufficient availability of antiviral drugs and vaccines against the arbovirus, the main approach for controlling mosquito-borne diseases is still through vector control.

*Bacillus thuringiensis* (Bt) is widely used as a biological control agent for pest control management and public health [11,12,13]. Recently, about one hundred Bt subspecies have been reported. Among them, *B. thuringiensis* subsp. *israelensis* (Bti) is widely used for mosquito control because of high toxin production [14,15]. The key steps for formation of Cry toxin pores in the plasma membrane of midgut cells that cause cell death include the following: (1) protoxin solubilization, (2) protoxin proteolytic activation by specific proteases, (3) interaction between active toxins and putative receptors, (4) oligomerization of toxins, and (5) insertion of toxin oligomers to epithelial cells [12,13]. The use of Cry toxins to kill insect pests mainly depends on the interaction between Cry toxins and important toxin receptors, such as alkaline phosphatase (ALP), aminopeptidase-N (APN), ATP-binding cassette (ABC) transporters, and cadherin (CAD) [12,16,17,18,19]. Other midgut proteins may interfere with the interactions between Cry toxins and toxin receptors to modulate the toxicity of Cry toxins. *A. aegypti* galectin-14 was recently found to compete with Cry11Aa for binding to ALP1 to alter the toxicity of Cry toxins, and galectin-6 also interacted with ALP1 to affect Cry toxicity (unpublished results). Therefore, it is important to understand the mechanism and interaction of other midgut proteins with Cry toxins and toxin receptors.

C-type lectins (CTLs) are carbohydrate-recognition proteins that play important roles in the innate immunity system [20]. CLTs have been identified in many plants, invertebrates, and vertebrates as carbohydrate recognition proteins [21,22]. CTLs usually contain one or two carbohydrate recognition domains (CRDs), which are composed of β-sheets, α-helices, and loops [23]. Furthermore, the specific motifs, such as Gln-Pro-Asp (QPD) and Glu-Pro-Asn (EPN), in the carbohydrate recognition domains are important for binding to galactose and mannose, respectively [24]. Insect CTLs can serve as pattern recognition receptors to enhance melanization and haemocyte encapsulation in *Manduca sexta* and *Drosophila melanogaster* [25,26], and stimulate phagocytosis of bacteria in *Anopheles gambiae* [27]. Additionally, the CTL expression level is affected by bacterial, viral, and fungal infections in many insects, such as *Helicoverpa armigera* [28]. Mosquitoes completely depend on the innate immune system to fight against pathogens because of the lack of the acquired immune system [29,30,31,32]. Therefore, the identification of mosquito immune-related genes/proteins, such as CTLs, is very important to better understand the mosquito defense mechanisms [33,34,35,36].

In the current study, we like to know whether immune-related proteins also play a role in Cry toxin tolerance and mainly focus on the interaction of *A. aegypti* midgut proteins with Cry11Aa and toxin receptors. *A. aegypti* CTL-20 was cloned, recombinant CTL-20 was expressed and purified in this study. Then, interactions of CTL-20 with Cry11Aa and ALP1 were confirmed by the Far-Western blot and ELISA methods. Furthermore, CTL-20 bound to *A. aegypti* larval brush border membrane vesicles (BBMVs), and the survival rate of *A. aegypti* larvae fed with Cry11Aa mixed with CTL-20 was significantly increased compared with the control larvae fed with Cry11Aa mixed with thioredoxin. Our novel results suggest that midgut proteins like CTLs may interfere with interactions between Cry toxins and toxin receptors by binding to both Cry toxins and receptors to alter Cry toxicity.

## 2. Results

### 2.1. Characterization of A. aegypti CTL-20

Based on the designed gene-specific primers, *A. aegypti* lectin gene CTL-20 (Accession no.: XP 001661644.2), containing predicted C-type lectin carbohydrate-recognition domain, was amplified by polymerase chain reaction (PCR) and the amplified fragment was cloned and sequenced. Sequence analysis indicated that the open reading frame of CTL-20 was 471 bp, encoding a protein of 156 amino acids with calculated molecular weight of 18 kDa and isoelectric point (*p*I) of 5.37.

**Figure 1:**
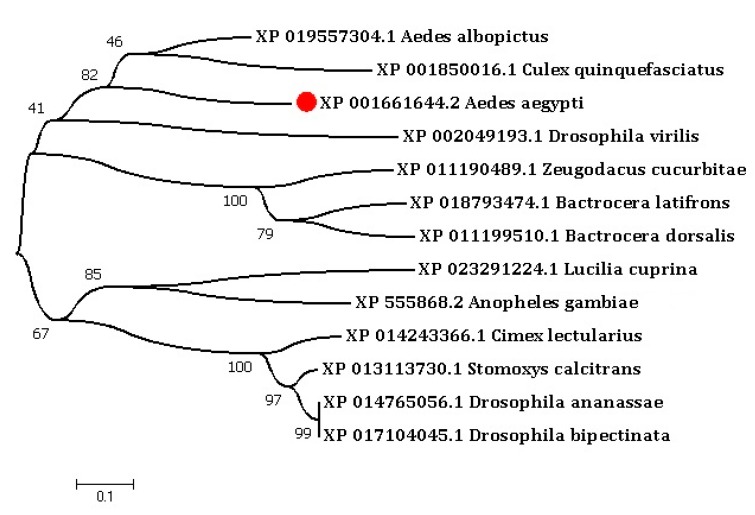
Phylogenetic tree of *A. aegypti* C-type lectin-20 (CTL-20) (XP 001661644.2) and homologous CTLs in other species. The evolutionary history was inferred using the neighbor-joining method. The bootstrap test values from 1000 replication are provided at each node. The evolutionary distances were computed using the p-distance method. The genus and species names were shown to the right of branches, respectively.

To determine the phylogenetic relationship of CTL-20 with homologous CTLs, CTLs from several insect species, including flies and mosquitoes, were identified and a phylogenetic tree was constructed. The result revealed that CTL-20 of *A. aegypti* was closely clustered with Culex mosquito CTLs (Figure 1).

### 2.2. CTL-20 Binds to ALP 1 and Cry11Aa

**Figure 2:**
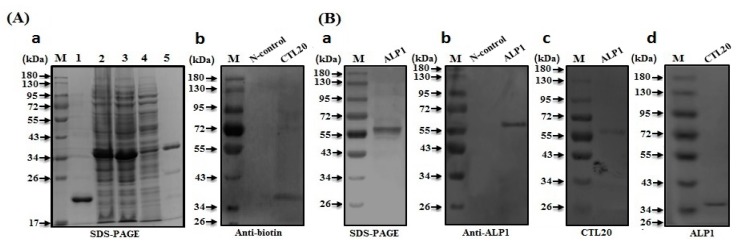
Interaction between recombinantCTL-20 and alkaline phosphatase (ALP)1 proteins. (**A**) SDS-PAGE and Western blot analysis of CTL-20 recombinant protein. (**a**). Lane M: protein marker; lanes 1–5:Purified recombinant thioredoxin (Trx) (control), CTL-20-Trx total proteins, supernatant of the total proteins, flow-through after binding to the resins, purified CTL-20-Trx recombinant protein, (**b**). The purified CTL-20-Trx protein detected with the corresponding polyclonal antibodies. (**B**) SDS and Western blot analysis of ALP1 recombinant protein. (**a**). Purified recombinant ALP1 protein; (**b**). The purified ALP1 protein detected with the corresponding polyclonal antibody; (**c**). Recombinant CTL-20-Trx fusion protein interacts with ALP1 by Far-Western blot analysis. ALP1 band was detected by antibody specific to CTL-20 after the membrane was probed with CTL-20-Trx; (**d**). CTL-20-Trx band was detected by antibody specific to ALP1 after the membrane was probed with ALP1.

**Figure 3:**
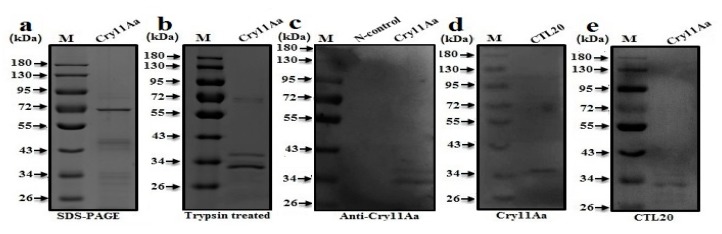
Interaction between recombinant CTL-20 and Cry11Aa proteins. (**a**). SDS-PAGE analysis of purified Cry11Aa protoxin; (**b**). The invitro processing of the Cry11Aa protoxin with trypsin (1:20 *w/w*); (**c**). Western blot analysis of cleaved fragments of Cry11Aa protoxin with anti-Cry antibody; (**d**). Trypsin-activated Cry11Aa binds to CTL-20-Trx protein and CTL-20-Trx was detected in the membrane by anti-Cry antibody when the membrane was probed with Cry11Aa; (**e**). Similarly, Cry11Aa fragments were detected by antibody specific to CTL-20 when the membrane was probed with recombinant CTL-20-Trx.

The use of Cry toxins against insect pests depends upon the interaction between Cry toxins and putative receptors, such as alkaline phosphatase (ALP), aminopeptidase-N (APN), and ATP-binding cassette (ABC) transporters [12,16,17,18,19]. However, if some third-party proteins could interfere with the interaction between Cry toxins and receptors, toxicity of Cry would be altered [37]. In order to test whether CTL-20 binds to Cry and/or putative receptors, recombinant CTL-20 and ALP1, as well as Cry11Aa protoxin, were expressed and purified in this study (Figure 2(A-a),(B-a), and Figure 3a). Western blot analysis confirmed the purified recombinant CTL-20-Trx and ALP1 proteins with ~37 kDa and ~65 kDa, and Cry11Aa fragments of ~32 and ~36 kDa detected by specific polyclonal rabbit antibodies to CTL-20 and ALP1 (Figure 2(A-b),(B-b)), and Cry11Aa (Figure 3c). Furthermore, the 37-kDa CTL-20-Trx fusion protein in the membrane was detected by biotin specific antibody when probed with biotinylated ALP1 (Figure 2(B-d)), and the ~65 kDa ALP1 receptor protein band was detected in the membrane by CTL-20 specific antibody when probed with biotinylated CTL-20-Trx (Figure 2(B-c)). These results suggest that CTL-20 can interact with ALP1 receptor. Interestingly, the CTL-20-Trx in the membrane was also detected by the biotin specific antibody when probed with biotinylated Cry11Aa (Figure 3d). Furthermore, the cleaved fragments of Cry11Aa (32 and 36 KDa) were detected in the membrane by CTL-20 specific antibody when probed with biotinylated CTL-20-Trx (Figure 3e). Together, these results suggest that CTL-20 can bind not only to ALP1, one of the Cry receptors, but also to Cry11Aa toxin.

### 2.3. CTL-20 Binds to BBMVs and ALP1

**Figure 4:**
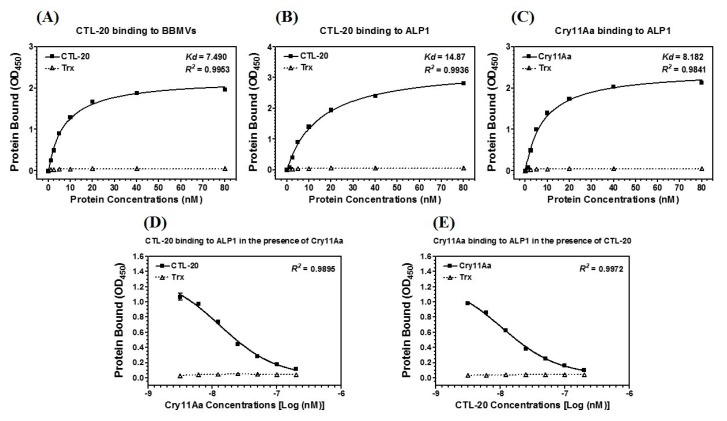
Binding of CTL-20 with brush border membrane vesicles (BBMVs) and ALP1, and binding of Cry11Aa with ALP1;(**A**). Binding of purified recombinant CTL-20-Trx and thioredoxin (Trx) to immobilized BBMVs; (**B**). Binding of purified recombinant CTL-20-Trx and thioredoxin (Trx) to immobilized ALP1; (**C**). Binding of trypsin-activated biotinylated Cry11Aa protein and thioredoxin to immobilized ALP1; (**D**). Binding of purified recombinant biotinylated CTL-20-Trx to immobilized ALP1 in the presence of increasing concentrations of unlabeled Cry 11Aa; (**E**). Binding of biotinylated Cry11Aa to immobilized ALP1 in the presence of increasing concentrations of unlabeled CTL-20. The solid lines represent nonlinear regression calculations of a one-site binding (**A**–**C**) or nonlinear regression calculations of a one-site competition binding (**D**,**E**).

To further confirm binding of CTL-20 to midgut BBMVs of *A. aegypti* larvae, ELISA binding assays were performed. We found that cumulative quantities of recombinant CTL-20-Trx protein bound to the coated BBMVs increased when higher concentrations of the fusion protein were applied, with a calculated *Kd* of 7.49 nM for one-site binding (Figure 4A). However, no significant binding activity was observed between the control thioredoxin (Trx) protein and BBMVs even at 80 nM of thioredoxin protein. Furthermore, we also determined binding of biotinylated CTL-20-Trx binding to ALP1, and the result showed that more CTL-20-Trx bound to immobilized ALP1 when increasing concentrations were applied, with a calculated *Kd* of 14.87 nM for one-site binding (Figure 4B). As a comparison, binding of Cry11Aa to immobilized ALP1 was also performed, with a calculated *Kd* of 8.182 nM for one-site binding (Figure 4C). These results suggest that CTL-20 has a higher affinity than Cry11Aa for ALP1. We also performed competitive binding assays to test whether CTL-20 and Cry11Aa bind to the same site in ALP1, and the results showed that unlabeled Cry11Aa competed with biotin-labeled CTL-20 for binding to ALP1 (Figure 4D) and unlabeled CTL-20 also competed with biotin-labeled Cry11Aa for binding to ALP1 (Figure 4E). These results suggest that CTL-20 and Cry11Aa bind to the same region in the ALP1.

### 2.4. CTL-20 Inhibits Larvicidal Activity of Cry Toxins

**Figure 5:**
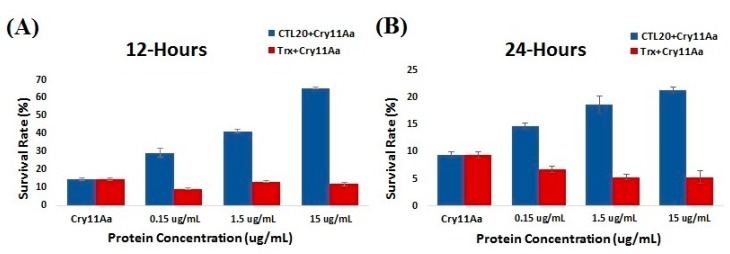
Bioassay of Cry11Aa in the presence of recombinant CTL-20 or thioredoxin protein. *A. aegypti* larvae were fed with purified Cry11Aa (0.85 μg/mL) mixed with increasing concentrations of recombinant thioredoxin (a control protein) or recombinant CTL-20-Trx protein (0.15, 1.5, 15 μg/mL), and the survival of mosquito larvae was recorded from 12 h (**A**) and 24 h (**B**) after feeding with three replicates.

To test whether the activity of Cry toxins could be changed when CTL-20 protein concentration was increased, bioassay experiments were performed. Mosquito larvae were fed Cry11Aa mixed with the purified recombinant CTL-20-Trx protein or thioredoxin protein as a control and the cumulative survival of larvae was recorded. The bioassay results clearly showed that the survival rate of mosquito larvae was significantly increased when CTL-20-Trx fusion protein was mixed with Cry11Aa pure protein as compared with the control larvae fed on Cry11Aa mixed with thioredoxin protein at 12 h (Figure 5A) and 24 h post-feeding (Figure 5B). These results suggest that CTL-20 can alter the larvicidal activity of Cry11Aa toxin against *A. aegypti* larvae.

## 3. Discussion

Bti produces different toxins such as Cry4Aa, Cry4Ba, Cry10Aa, Cry11Aa, and the Cyt family toxins (Cyt1Aa and Cyt2Ba), which were used widely throughout the world for mosquito control and management [11,12,13,38]. Among these toxins, Cry11Aa was the most promising for mosquitocidal activity [39]. The interaction of Cry11Aa with important toxin receptors such as ALP in the midgut cells causes toxicity to *A. aegypti* larvae [17], as interaction of active toxins with their membrane receptors is the key step for the molecular mechanism of pore formation [37]. Toxicity of Bt will thus be greatly altered if the key interaction between toxins and their receptors is diminished or even eliminated. Recently, it has been reported that *A. aegypti* galectin-14 can bind to BBMVs and ALP1, and may compete with Cry11Aa for binding to ALP1; feeding recombinant galectin-14 can significantly increase the survival of mosquito larvae treated with Bti toxins (unpublished results). *A. aegypti* galectin-6 also interacted with ALP1 to affect the Cry toxicity (unpublished results).Therefore, detection and identification of midgut proteins that can interfere with this key step may open up a new research direction to fully understand Bt mechanisms and provide great theoretical basis for the development of new biopesticides for mosquito control.

In current study, CTL-20 was found to inhibit Cry toxicity by apparently neutralizing Cry11Aa and blocking Cry11Aa from binding to ALP1 receptor. It has been reported that the CTL-20 was up regulated in Bti tolerant *A. aegypti* strains [40,41]. Thus, increased expression of CTL-20 in *A. aegypti* larvae may partially account for the Bti tolerance. Carbohydrate-recognition domains of lectins and Cry toxin domain III adopt similar β-sandwich fold, and similar structure to the β-prism structure of Cry toxin domain II [42,43,44], which may explain why CTL-20 and Cry11Aa both bound to ALP1. Competitive binding assays suggest that the same region in ALP1 is involved in interaction with CTL-20 and Cry11Aa.Bioassay results clearly showed that survival rate of mosquito larvae was significantly increased, when fed Cry11Aa toxin mixed with CTL-20-Trx fusion protein compared with larvae fed on Cry11Aa toxin mixed with the thioredoxin control protein. These results indicated that with more CTL-20 added, CTL-20 can neutralize certain amounts of Cry11Aa, at the same time, CTL-20 binds to ALP1 and blocks Cry11Aa from binding to ALP1. The combined effect is the significant reducing of Cry11Aa binding to BBMVs, resulting in significantly reduced toxicity against *A. aegypti* larvae.

Our findings are useful and will provide a sound base for further study of interactions between midgut proteins and Cry toxins and/or toxin receptors. After all, there might be many midgut proteins, which may interfere with interactions between Cry toxins and different toxin receptors, thus altering the toxicity of Cry toxins. Future research will investigate expression of CTL-20 in Bti susceptible and tolerant mosquito strains, as well as regulation of CTL-20. Compounds/chemicals that can counterpart the effect of CTL-20 in the midgut may be good additives to increase the toxicity of Cry toxins for mosquito control. These studies will not only greatly enrich mechanism of Bt, but also provide great value for mosquito and transmitted disease control.

## 4. Materials and Methods

### 4.1. Mosquitoes and Sample Collection

*A. aegypti* was maintained in laboratory and reared under optimal conditions of 28 °C and 70–80% relative humidity with a 14 h light and 10 h dark photoperiod. The recombinant Bt strain pCG6 producing Cry11Aa and with high toxicity against mosquitoes was provided by Dr. Sarjeet R. Gill laboratory at University of California, Riverside, CA, USA. *Escherichia coli* (*E. coli*) strain DH5α and BL21 (DE3) were purchased from TaKaRa (Dalian, China). Plasmid pMD18-T (TaKaRa, Dalian, China) and pET32a vector used in this study were stored in our lab. EZ-Link-NHS-Biotin was purchased from Thermo Fisher Scientific (Waltham, MA, USA). Expression vectors for recombinant *A. aegypti* ALP1 were obtained from Dr. Sarjeet R. Gill laboratory. Biotin specific antibody Streptavidin/AP was purchased from BIOSS antibodies (Beijing, China). Streptavidin horse-radish peroxidises (HRP) conjugate antibody was purchased from Beyotime biotech (Shanghai, China), Anti-6xHis antibody and rabbit polyclonal antibodies were from BBI life science (Shanghai, China). *ProteinIso^®^* Ni-NTA resins were obtained from TransGen Biotech (Beijing, China).

### 4.2. Cry11Aa Toxin Purification and Activation

*B. thuringiensis* strain (pCG6), producing Cry11Aa toxin [45], was grown in nutrient broth sporulation medium containing erythromycin (25 mg/mL) at 30 °C for 4–5 days up to complete sporulation [46]. The spores and crystal inclusions were harvested and purified according to the procedure described by previously reported studies [47,48,49]. The purified Cry11Aa inclusions were activated by trypsin (1:20, *w/w*) at 37 °C [50]. The activated Cry11Aa was biotinylated and purified using a Sephadex G25 column according to the manufacturer’s instruction (GE Healthcare Life Science, Pittsburgh, PA, USA).

### 4.3. CTL-20 Full-Length cDNA Cloning

*A. aegypti* cDNA sequence (GeneID: AAEL011407) to encode a ‘CTL’ domain containing protein was identified using VectorBase (http://www.vectorbase.org). The coding sequence was amplified by polymerase chain reaction (PCR) with designed gene-specific forward primer with *Nco1* restriction site (5′CATGCCATGGTTGGAG CAACTTCA3′) and reverse primer with the *Hind111* site (5′CCCAAGCTTATCCAAGATGCAACTCCTA3′). The purified PCR product contained DNA fragment was cloned in PMD-18T vector and confirmed by sequencing. The recombinant plasmid was digested with *Nco1*/*Hind111* and ligated to the pET-32a (+) expression vector. Transformation preceded using competent *E. coli* BL21 (DE3) cells for expression of recombinant CTL-20 protein.

### 4.4. Bioinformatics Analysis

DNAMAN (Lynnon Corporation, Quebec, QC, Canada) software was used to analyze the homology of deduced protein sequence of CTL-20. The protein size and amino acid properties were determined by using ProtParam tool (Swiss Institute of Bioinformatics, Basel, BSL, Switzerland; http://web.expasy.org/prot param/). The CRD domains were examined by using SMART tool software (version.8.0, EMBL, Meyerhofstrasse 1, 69117 Heidelberg, HD, Germany; http://smart.embl-heidelberg.de/). The orthologous protein sequences were identified through BLASTP search using NCBI database (http://www.ncbi.nlm.nih.gov/). The protein sequences were aligned by the MUSCLE module of the MEGA6 software (version 6.0, Research center for Genomics and Bioinformatics, Tokyo Metropolitan University, Tokyo, TYO, Japan). A neighbor-joining tree was generated with a bootstrap of 1000 replications [51].

### 4.5. Prokaryotic Expression and Purification of Recombinant CTL-20

A single colony from BL21 cells was picked and inoculated in LB media with specific antibiotic (0.1% *Ampicillin*) in constant shaking incubator at 37 °C until OD_600_ reached 0.6–0.8. Induction was preceded by 0.5–1 mM isopropyl-β-d-1-thiogalactopyranoside (IPTG) for 20 h at 16 °C with constant agitation of 200 rpm. Bacteria were collected, washed, and sonicated in binding buffer (pH 7.4). Soluble proteins were collected in supernatant and run through Ni-NTA slurry (TransGen Biotech, Beijing, China) and purified by following the manufacturer’s instructions. Purified recombinant proteins were separated on 12% SDS-PAGE gels and stained with Coomassie brilliant blue R250. Recombinant thioredoxin (Trx) as a control protein and His tagged recombinant protein ALP1 were also purified by Ni-NTA chromatography.

### 4.6. Insect Feeding Assay

Bioassays were assessed to check the susceptibility of these mosquito larvae after feeding Cry11Aa pure toxin protein with LC_50_ of 0.85 μg/mL. For this purpose, fourth instar *A. aegypti* larvae (25 larvae/cup) were fed with Cry11Aa (0.85 μg/mL) mixed with recombinant CTL-20-Trx or thioredoxin (control) protein (0, 0.15, 1.5, 15 μg/mL) in 30 mL dechlorinated water, and survival rates were recorded from 12 to 24 h. Each treatment experiment was repeated three times with three replicates.

### 4.7. Preparation of A. aegypti BBMVs

About 1000 midguts were collected from the late fourth instar *A. aegypti* larvae. BBMVs were prepared by MgCl_2_ differential precipitation modified method as described by Carroll & Ellar (1993) [52]. Protein concentration was determined by the Bradford method (1976) with BSA as a standard using the Bio-Rad protein assay and stored at −80 °C until use [53].

### 4.8. Western Blot and Far-Western Blot Analyses

To detect the recombinant proteins expression by Western blot analysis, the purified recombinant *A. aegypti* CTL-20, ALP, and Cry11Aa were separated on 10% SDS-PAGE and then transferred to Polyvinylidene difluoride (PVDF) membranes. The membrane was blocked with PBSM (PBS + 0.05% skim milk) for 2 h at 37 °C incubator or overnight at 4 °C, washed with PBST (PBS + 0.05% Tween-20) and incubated with primary rabbit polyclonal antibody (1:3000) to each protein and bind with goat anti-rabbit antibody (1:3000) as secondary antibody. Antibody binding was visualized by a color reaction catalyzed by conjugated alkaline phosphatase as described previously [37].

Far-Western blot examination was performed based on the previously described method [37]. To confirmed interaction of target proteins, purified recombinant *A. aegypti* ALP1, CTL-20-Trx, and Cry11Aa were separated on 10% SDS-PAGE and transferred to PVDF membrane. The membrane was blocked with 5% dry skim milk in PBS for 2 h at 37 °C and then probed with purified biotinylated recombinant CTL-20, ALP1, or Cry11Aa in PBS (pH 7.4) containing 0.1% BSA overnight at 4 °C with gentle rocking and washed the membrane with PBST and PBS buffer. The membrane was then incubated with biotin specific antibody Streptavidin/AP (1:3000) for 2 h. After washing many times with PBST and PBS buffer, the binding of interacting proteins was checked using BCIP/NBT alkaline phosphatase color development kit (Beyotime biotech, Shanghai, China).

### 4.9. Plate ELISA Assays

The binding of CTL-20-Trx fusion protein to BBMVs and ALP1, as well as Cry11Aa to ALP1, was performed by plate ELISA assays as previously described [37]. The purified recombinant CTL-20-Trx, Cry11Aa, and thioredoxin were biotinylated with EZ-Link-NHS-Biotin following the manufacturer’s instructions (ThermoFisher Scientific, Waltham, MA, USA). Ninety-six-well ELISA plates FEP-100-008, Jet Biofil, (Guangzhou Jet Bio-Filtration Co. Ltd., Guangzhou, China) were coated with 4 μg/well BBMVs, recombinant ALP1, Cry11Aa, or CTL-20-Trx, and kept overnight at 4 °C. Only ELISA buffer (Na_2_CO_3_, pH 9.6) was added in uncoated wells as negative control and the plate was washed with PBS buffer (pH 7.4) three times. Afterwards, increasing concentrations of biotinylated CTL-20-Trx, Cry11Aa, or thioredoxin (0–80 nM) were supplied to each well of the plate and further incubated for 2 h at 37 °C. Meanwhile, 10 nM of recombinant biotin-labeled CTL-20 with increasing concentration of unlabeled Cry11Aa (0–200 mM) and biotin-labeled Cry11Aa (10 nM) with unlabeled CTL-20 (0–200 nM) mixture or thioredoxin (0–200 nM) were also coated to plate well for competitive binding assays to test whether CTL-20 and Cry11Aa could bind to the same site in ALP1. The plates were washed with PBS buffer (pH7.4) three times, the biotinylated bound protein was detected by incubation of the plates with streptavidin horse-radish peroxidase (HRP) conjugate antibody (1:3000) based on the manufacturer’s instruction. After washing three times with PBST buffer (PBS + 0.1% Tween-20) and three times with PBS buffer, the chromogenic reagent kit EL-TMB P0209 (Beyotime Biotech, Jiangsu, China) was used for development of color. Finally, the absorbance was checked at 450 nm on the Multiskan™ GO Microplate Spectrophotometer, (Thermo 180 Scientific, Waltham, MA, USA).
